# Supplementary material for: Polyamine-mediated ferroptosis amplification acts as a targetable vulnerability in cancer
Source: Nat Commun. 2024 Mar 19;15:2461. doi: 10.1038/s41467-024-46776-w (PMC10951362; doi:10.1038/s41467-024-46776-w)

Supplementary Information

Polyamine-mediated ferroptosis amplification acts as a targetable vulnerability in cancer

Table of contents

Supplementary Figure 1-4

Uncropped blots in supplementary figures

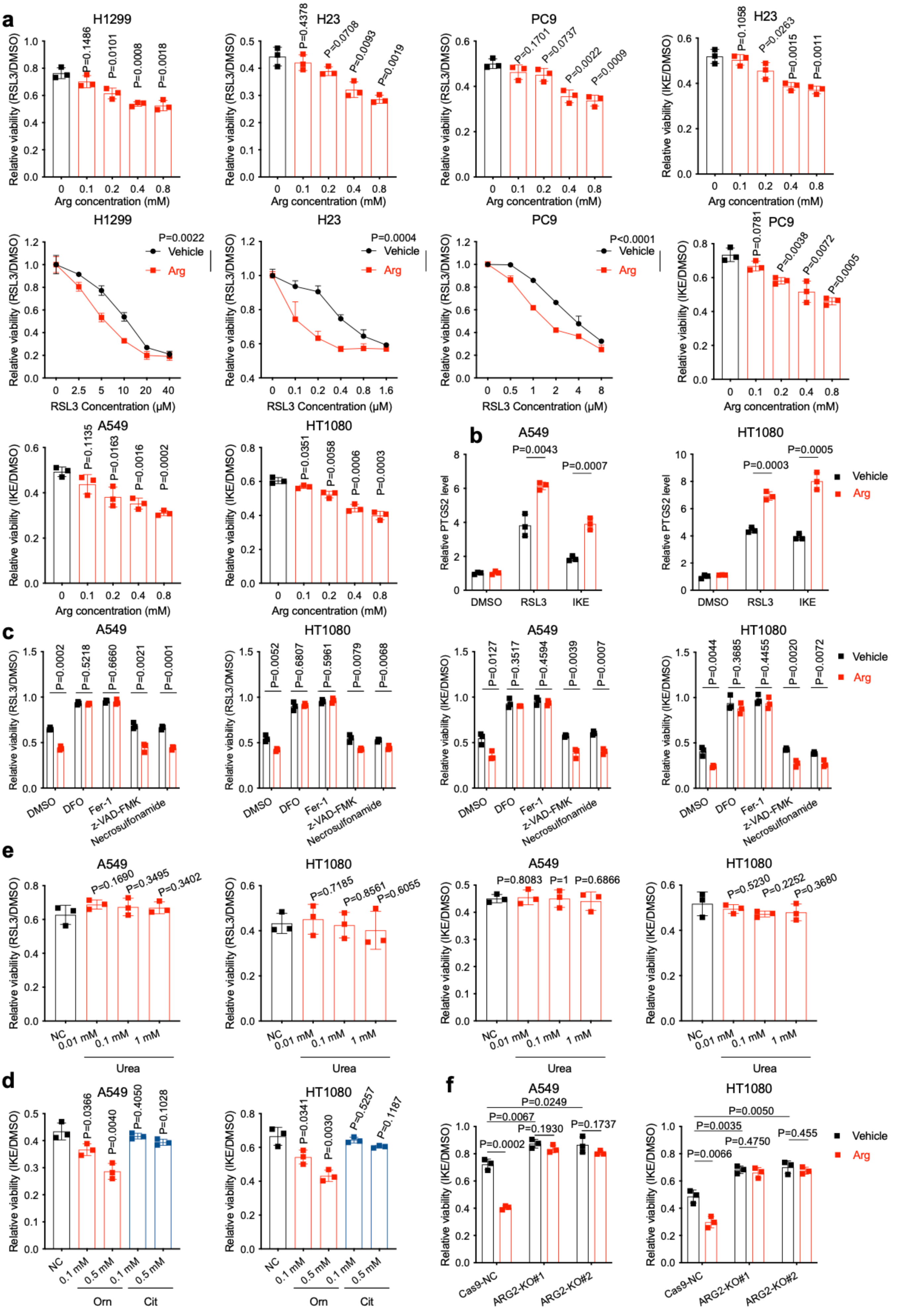

Supplementary Figure 1. Arginine and ornithine are ferroptosis promoters. a, Cell viability in A549, HT1080, H1299,

H23, or PC9 cells treated with RSL3 (H1299: 40 μM for 24 h; H23: 0.2 μM for 6 h; PC9: 0.5 μM for 6 h; or as indicated) or IKE (A549: 40 μM for 48 h; HT1080: 4 μM for 12 h; H23 and PC9: 2 μM for 18 h) following pre-treatment with arginine. **b**, mRNA levels of PTGS2 in A549 or HT1080 cells treated with DMSO, RSL3 (A549: 0.5 uM; HT1080: 0.05 μM) or IKE (A549: 20 μM; HT1080: 0.5 μM) for 8 h following pre-treatment with 0.5 mM arginine for 4 h, determined by qPCR. **c**, Cell viability in A549 or HT1080 cells treated with RSL3 or IKE combined with or without DFO, Fer-1, z-VAD-FMK, or necrosulfonamide following pre-treatment with arginine. **d**, Cell viability in A549 or HT1080 cells treated with IKE following pre-treatment with ornithine or citrulline as indicated. **e**, Cell viability in A549 or HT1080 cells treated with RSL3 or IKE following pre-treatment with urea as indicated for 4 h. **f**, Cell viability in A549 or HT1080 cells with indicated genotypes treated with IKE following pre-treatment with arginine. Data are presented as the mean ± SD, n = 3 independent experiments. Unpaired two-tailed Student's t tests are used. Source data are provided as a Source Data file.

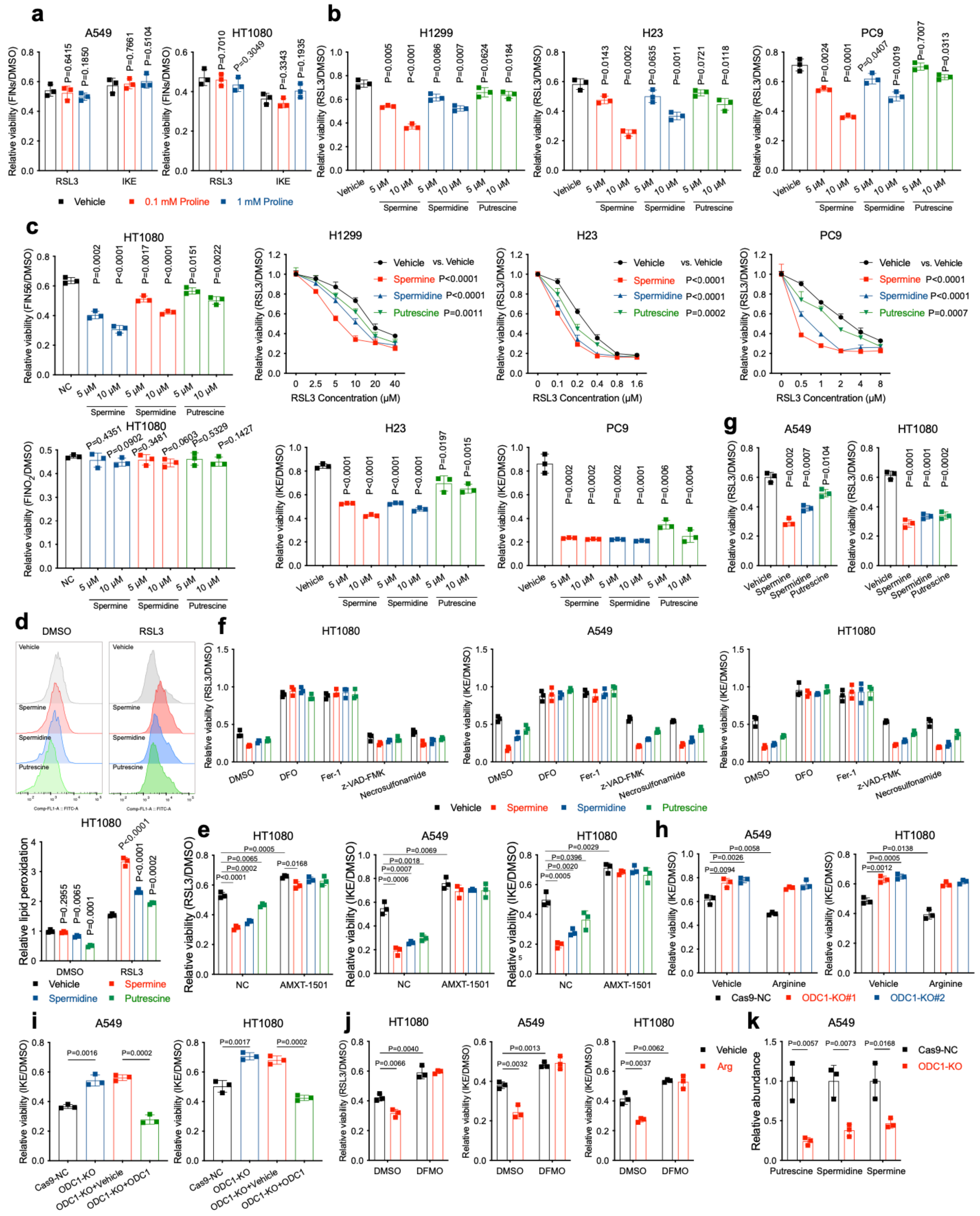

Supplementary Figure 2. Polyamines are potent ferroptosis mediators. a, Cell viability in A549 or HT1080 cells treated with RSL3 or IKE following pre-treatment with proline as indicated for 4 h. **b**, Cell viability in H1299, H23, or PC9 cells treated with RSL3 or IKE following pre-treatment with polyamines. **c**, Cell viability in HT1080, H1299, H23, PC9 cells treated with FINs (HT1080: 10 μM FIN56/$FINO_2$ for 12 h) following pre-treatment with polyamines. **d**, Lipid peroxidation in HT1080 cells treated with 0.25 μM RSL3 for 3 h following pre-treatment with polyamines. **e**, Cell viability in A549 or

HT1080 cells treated with RSL3 or IKE following pre-treatment with AMXT-1501 and polyamines. **f**, Cell viability in A549 or HT1080 cells treated with RSL3 or IKE combined with or without DFO, Fer-1, z-VAD-FMK, or necrosulfonamide following pre-treatment with polyamines. **g**, Cell viability in A549 or HT1080 cells treated with RSL3 following pre-treatment with 10 μM polyamines and 1 mM aminoguanidine for 4 h. **h**, Cell viability in A549 or HT1080 cells with indicated genotypes treated with IKE following pre-treatment with arginine. **i**, Cell viability in A549 or HT1080 cells with indicated genotypes treated with IKE. **j**, Cell viability in A549 or HT1080 cells treated with RSL3 or IKE following pre-treatment with DFMO and arginine. **k**, Relative abundance of polyamines in Cas9-NC and ODC1-KO A549 cells determined by LC-MS. Data are presented as the mean ± SD, n = 3 independent experiments. Unpaired two-tailed Student's t tests are used. Source data are provided as a Source Data file.

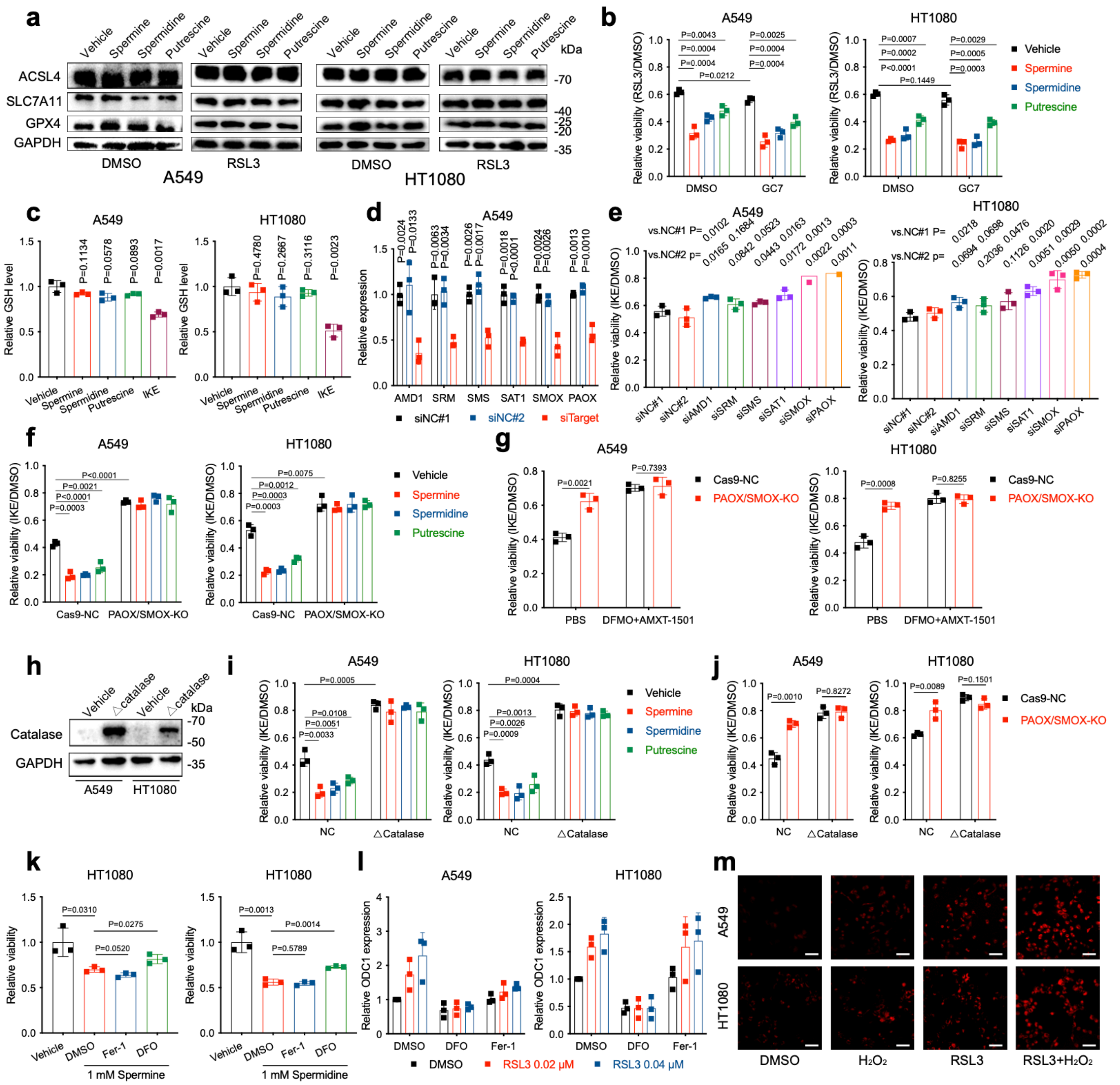

Supplementary Figure 3. Polyamines do not interfere with known ferroptosis regulators. a, Protein levels of ACSL4, SLC7A11, and GPX4 in A549 or HT1080 treated with DMSO or RSL3 following pre-treatment with polyamines. **b**, Cell viability in A549 or HT1080 cells treated with RSL3 following pre-treatment with 1 µM GC7 for 16 h and polyamines. **c,** Glutathione levels in A549 or HT1080 cells treated with 10 µM polyamines or IKE (A549: 20 µM; HT1080: 2 µM) for 8 h. **d,** mRNA levels of the target genes in A549 cells transfected with corresponding siRNAs, determined by qPCR. **e,** Cell viability in A549 or HT1080 cells treated with IKE following siRNAs transfection for 48 h. **f,** Cell viability in Cas9-NC and PAOX/SMOX-KO A549 or HT1080 cells treated with IKE following pre-treatment with AMXT-1501 and DFMO. **g**, Cell viability in Cas9-NC and PAOX/SMOX-KO A549 or HT1080 cells treated with IKE following pre-treatment with AMXT-1501 and DFMO. **h,** Protein levels of catalase in A549 and HT1080 with its overexpression. **i**, Cell viability in vehicle- and △catalase-overexpressed A549 or HT1080 cells treated with IKE following pre-treatment with polyamines. **j**, Cell viability in A549 or HT1080 cells with indicated genotypes treated with IKE. **k**, Cell viability in HT1080 cells treated with 1 mM

spermidine or spermine combined with or without DFO or Fer-1 for 12 h. **l**, Quantification of the protein expression level by ImageJ in Figure 4c. **m**, Confocal microscope images of FerroOrange-stained A549 or HT1080 cells treated with RSL3 (A549: 2 μM; HT1080: 0.25 μM) or H_2O_2 (20 μM) for 3 h (scale bars, 100 μm). Data are presented as the mean ± SD, n = 3 independent experiments. Unpaired two-tailed Student's t tests are used. Source data are provided as a Source Data file.

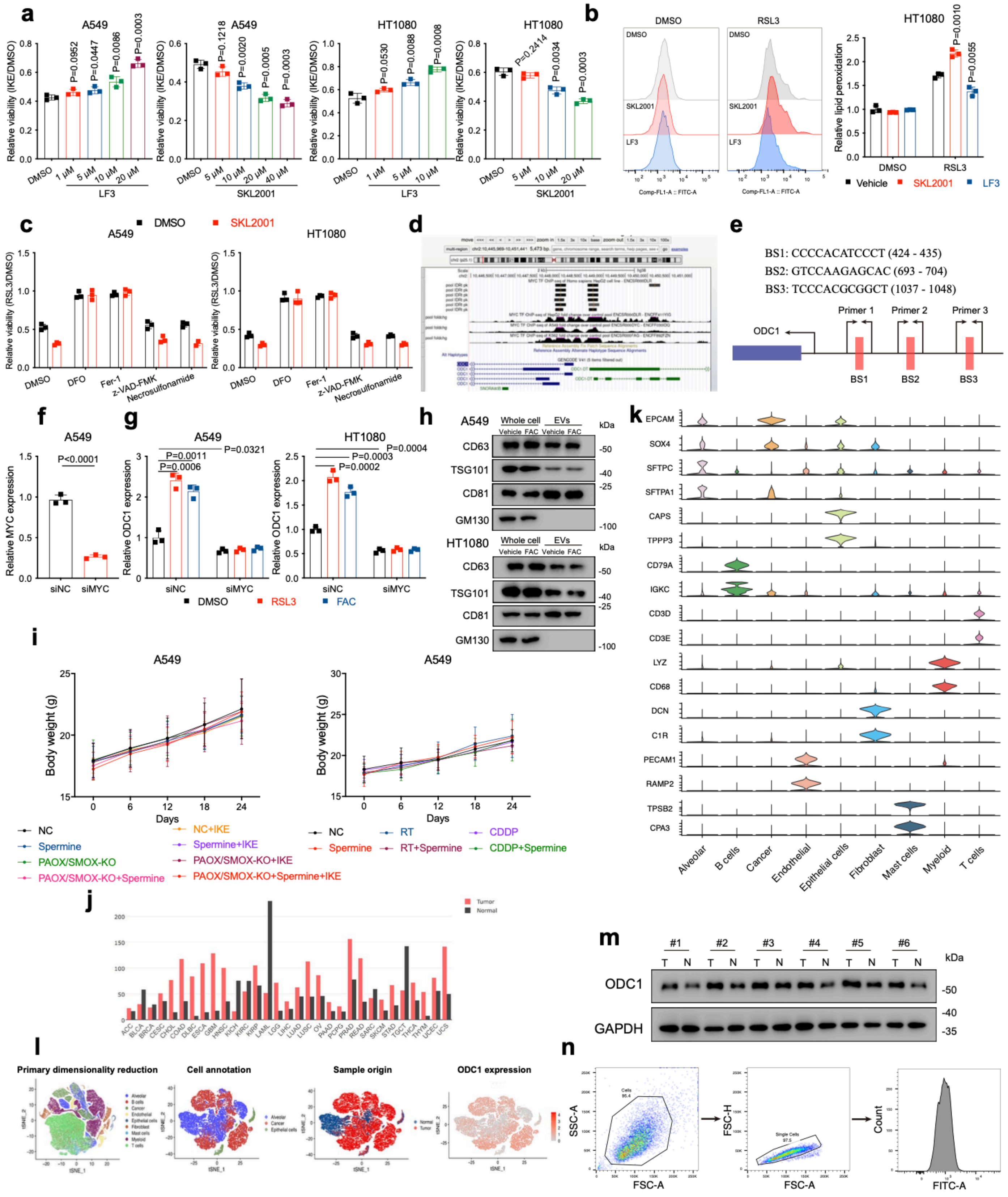

Supplementary Figure 4. WNT/MYC pathway mediates the ODC1 upregulation induced by iron-overload and promotes ferroptosis. a, Cell viability in A549 or HT1080 cells treated with IKE following pre-treatment with LF3 or SKL2001 as indicated. **b,** Lipid peroxidation in HT1080 treated with RSL3 following pre-treatment with LF3 or SKL2001. **c,** Cell viability in A549 or HT1080 cells treated with RSL3 combined with or without DFO, Fer-1, z-VAD-FMK, or necrosulfonamide following pre-treatment with SKL2001. **d,** MYC CHIP-Seq data from ENCODE database. **e,** The relative

positions and sequences of predicted binding sites (BS) of MYC in the promoter region of ODC1. The data was mined from the JASPAR database. **f**, mRNA levels of MYC in A549 cells transfected with corresponding siRNAs, determined by qPCR. **g,** mRNA levels of ODC1 in MYC-knockdown A549 or HT1080 cells treated with DMSO or RSL3, determined by qPCR. **h**, EVs were isolated from cell culture medium of A549 and HT1080 cells treated with PBS or FAC for 24 h. The cells and EVs were lysed and the protein levels of CD63, CD81, TSG101, and GM130 were determined by western blotting. **i**, The body weight growth of mice receiving different treatments as indicated. **j**, The relative expression level of ODC1 in different cancer types and corresponding normal tissues. The plotted data was mined from TCGA database and visualized using the GEPIA online tool. **k**, Violin plots depicting the expression of representative markers across each cell type identified in the malignant and non-malignant lung tissues. **l**, After primary dimensionality reduction using tSNE method, 44,715 alveolar, cancer, and epithelial cells were annotated based on the expression of known marker genes. The sample origin of these cells and the expressing pattern of ODC1 were visualized. **m**, The protein levels of ODC1 in the paired lung adenocarcinoma and adjacent normal lung tissue, determined by western blotting (n = 6). **n,** The gating strategy of BODIPY-C11 lipid peroxidation assay. Data are presented as the mean ± SD, n = 3 independent experiments. Unpaired two-tailed Student's t tests are used. Source data are provided as a Source Data file.

Supplementary Figure 3a. Uncropped scans.

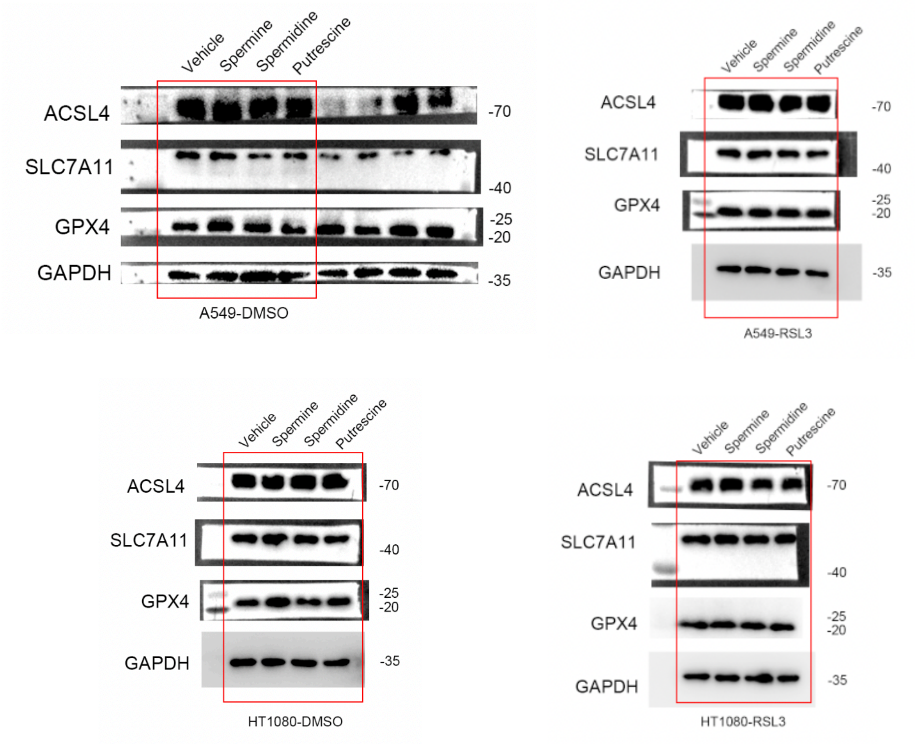

Supplementary Figure 3h. Uncropped scans.

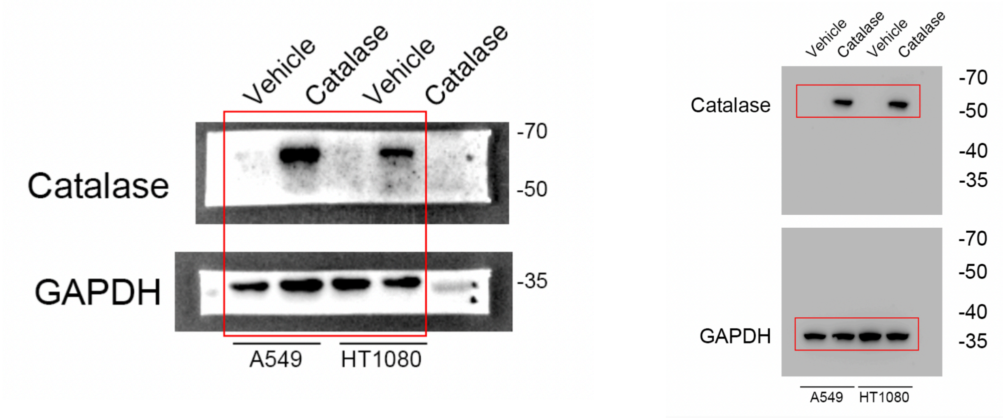

Supplementary Figure 4h. Uncropped scans.

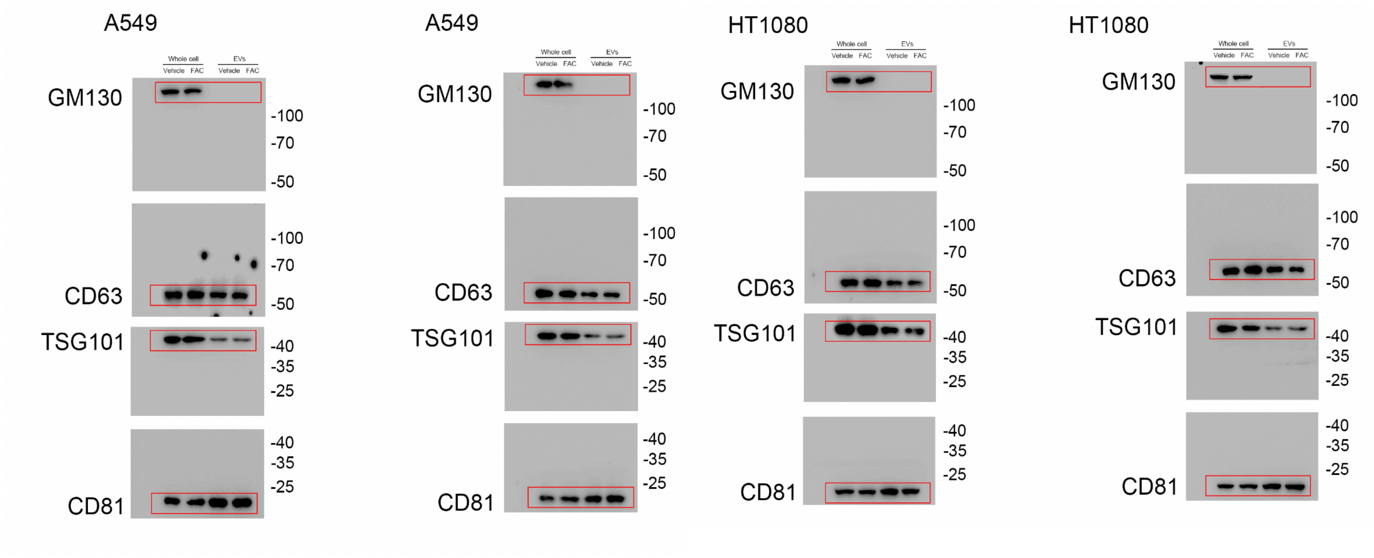

Supplementary Figure 4m. Uncropped scans.

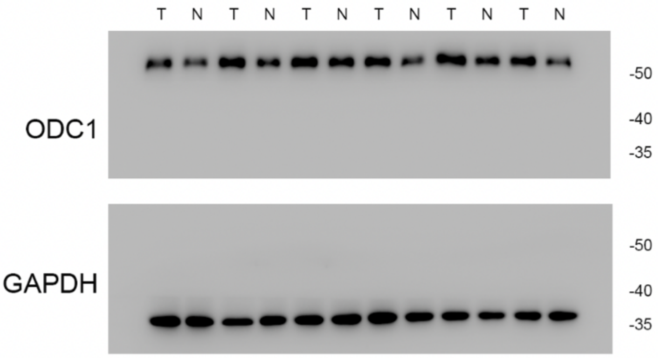

Supplement: Supplementary file 1 — Supplementary Information [file 41467_2024_46776_MOESM1_ESM.pdf]
