# Supplementary material for: Polyamine-mediated ferroptosis amplification acts as a targetable vulnerability in cancer
Source: Nat Commun. 2024 Mar 19;15:2461. doi: 10.1038/s41467-024-46776-w (PMC10951362; doi:10.1038/s41467-024-46776-w)
Supplement: Supplementary file 2 — Reporting Summary [file 41467_2024_46776_MOESM2_ESM.pdf]

nature portfolio

Corresponding author(s): Boyi Gan, Cheng Zhan

Last updated by author(s): Feb 23, 2024

Reporting Summary

Nature Portfolio wishes to improve the reproducibility of the work that we publish. This form provides structure for consistency and transparency in reporting. For further information on Nature Portfolio policies, see our Editorial Policies and the Editorial Policy Checklist.

Statistics

For all statistical analyses, confirm that the following items are present in the figure legend, table legend, main text, or Methods section.

n/a	Confirmed	
☐	☒	The exact sample size (*n*) for each experimental group/condition, given as a discrete number and unit of measurement
☐	☒	A statement on whether measurements were taken from distinct samples or whether the same sample was measured repeatedly
☐	☒	The statistical test(s) used AND whether they are one- or two-sided<br>*Only common tests should be described solely by name; describe more complex techniques in the Methods section.*
☒	☐	A description of all covariates tested
☒	☐	A description of any assumptions or corrections, such as tests of normality and adjustment for multiple comparisons
☐	☒	A full description of the statistical parameters including central tendency (e.g. means) or other basic estimates (e.g. regression coefficient) AND variation (e.g. standard deviation) or associated estimates of uncertainty (e.g. confidence intervals)
☐	☒	For null hypothesis testing, the test statistic (e.g. *F*, *t*, *r*) with confidence intervals, effect sizes, degrees of freedom and *P* value noted<br>*Give P values as exact values whenever suitable.*
☒	☐	For Bayesian analysis, information on the choice of priors and Markov chain Monte Carlo settings
☒	☐	For hierarchical and complex designs, identification of the appropriate level for tests and full reporting of outcomes
☐	☒	Estimates of effect sizes (e.g. Cohen's *d*, Pearson's *r*), indicating how they were calculated

Our web collection on statistics for biologists contains articles on many of the points above.

Software and code

Policy information about availability of computer code

Data collection	BD Accuri C6 and FACS Aria II were used to collect flow cytometry data. ExionLC™ System and QTRAP® 6500+ were used to collect liquid chromatography with tandem mass spectrometry (LC-MS) data.
Data analysis	GraphPad Prism software (7.0) was used for bar/line graphs output. Microsoft Excel was used for statistical analysis. FlowJo_V10 was used for flow data analysis. The RNA-Seq and LC-MS data were analyzed and visualized by R software (V4.0.5) with limma, ggplot2, pheatmap, and seruat packages. The softwares and algorithms for data analyses in this study are all well-established from previous work. There is no unreported algorithm used in this paper.

For manuscripts utilizing custom algorithms or software that are central to the research but not yet described in published literature, software must be made available to editors and reviewers. We strongly encourage code deposition in a community repository (e.g. GitHub). See the Nature Portfolio guidelines for submitting code & software for further information.

Data

Policy information about availability of data

All manuscripts must include a data availability statement. This statement should provide the following information, where applicable:

- Accession codes, unique identifiers, or web links for publicly available datasets
- A description of any restrictions on data availability
- For clinical datasets or third party data, please ensure that the statement adheres to our policy

The TCGA and CTRP publicly available data used in this study are available at https://xenabrowser.net/datapages/?cohort=GDC%20Pan-Cancer%20(PANCAN)

&removeHub=https%3A%2F%2Fxena.treehouse.gi.ucsc.edu%3A443, http://gepia.cancer-pku.cn/detail.php?gene=ODC1, and https://portals.broadinstitute.org/ctrp.v2.1/. The ENCODE data used in this study is available at https://www.encodeproject.org/experiments/ENCSR000DLR/. The scRNA-Seq publicly available data used in this study are available in the ArrayExpress database [accession numbers E-MTAB-6149 and E-MTAB-6653, https://www.ebi.ac.uk/biostudies/arrayexpress/studies/E-MTAB-6149, https://www.ebi.ac.uk/biostudies/arrayexpress/studies/E-MTAB-6653] and Human Cell Atlas Data Coordination Platform database [accession number PRJEB31843, https://www.ncbi.nlm.nih.gov/bioproject/?term=PRJEB31843]. The RNA-Seq data generated in this study have been deposited in the SRA database under accession code PRJNA979805 [https://www.ncbi.nlm.nih.gov/sra/?term=PRJNA979805]. The metabolomics data are deposited in the Figshare database under accession code 24037836 [https://doi.org/10.6084/m9.figshare.24037836.v3]. The processed data are available in Source data file. The remaining data are available within the Article, Supplementary Information or Source Data file. Source data are provided with this paper.

Research involving human participants, their data, or biological material

Policy information about studies with human participants or human data. See also policy information about sex, gender (identity/presentation), and sexual orientation and race, ethnicity and racism.

Reporting on sex and gender	The tumour and corresponding adjacent normal tissues were obtained from the patients diagnosed with LUAD who received surgery in the Department of Thoracic Surgery, Zhongshan Hospital, Fudan University between May 2018 and Apr 2023 (10 pairs, female 6, male 4). After quick-freezing with liquid nitrogen, the samples were stored at -80 ℃ before processing. Frozen human tissues were used for protein extraction-western blotting (6 pairs, female 4, male 2) and IHC staining (4 pairs, female 2, male 2).
Reporting on race, ethnicity, or other socially relevant groupings	All the LUAD patients included in this study who received surgery in the Department of Thoracic Surgery, Zhongshan Hospital, Fudan University are Chinese people, belonging to the yellow race.
Population characteristics	The 10 patients' sample were allocated randomly to the Western blot group and IHC group. The patients were diagnosed with LUAD and received surgery in the Department of Thoracic Surgery, Zhongshan Hospital, Fudan University between May 2021 and Mar 2023. In the Western blot group, the maximum age is 72, the minimum age is 47, the median age is 67. In the IHC group, the maximum age is 71, the minimum age is 52, the median age is 58.5.
Recruitment	All the patients were diagnosed with LUAD and received surgery in the Department of Thoracic Surgery, Zhongshan Hospital, Fudan University between May 2018 and Apr 2023.
Ethics oversight	The use of patient samples in this study was approved by the Ethics Committee of Zhongshan Hospital, Fudan University, China (Approval No: B2022-180R). All of the patients have provided written informed consent, and the study was conducted in accordance with the Declaration of Helsinki.

Note that full information on the approval of the study protocol must also be provided in the manuscript.

Field-specific reporting

Please select the one below that is the best fit for your research. If you are not sure, read the appropriate sections before making your selection.

☒ Life sciences ☐ Behavioural & social sciences ☐ Ecological, evolutionary & environmental sciences

For a reference copy of the document with all sections, see nature.com/documents/nr-reporting-summary-flat.pdf

Life sciences study design

All studies must disclose on these points even when the disclosure is negative.

Sample size	Sample size (n = 3 for most experiments and n = 6 or 7 for tumour xenograft assay) was chosen to ensure an adequate statistical power. All experiments were independently performed at least three times. Ref: Lee H, et al. Cell cycle arrest induces lipid droplet formation and confers ferroptosis resistance. Nat Commun. 2024 Jan 2;15(1):79.
Data exclusions	In the "Tumour xenograft experiment" section, mice that died before the end of the experiment were not included in the final analysis.
Replication	Multiple independent repeats were included for related experiments. Major findings were performed for at least three times to make sure similar results are reproducible.
Randomization	All cells and the animals were randomly allocated to experimental groups.
Blinding	For cell-based experiments, cell seeding and treatment (for example, DMSO or RSL3), measurement for cell viability, qPCR/WB, and data collection/analyzation, were conducted blindly. FACS, IHC, or photo capture were performed by different individuals who were blinded to the experimental groups. RNA-Seq and Mass spectrometry analysis were blinded prior to analysis.

Reporting for specific materials, systems and methods

We require information from authors about some types of materials, experimental systems and methods used in many studies. Here, indicate whether each material, system or method listed is relevant to your study. If you are not sure if a list item applies to your research, read the appropriate section before selecting a response.

Materials & experimental systems

n/a	Involved in the study
☐	☒ Antibodies
☐	☒ Eukaryotic cell lines
☒	☐ Palaeontology and archaeology
☐	☒ Animals and other organisms
☒	☐ Clinical data
☒	☐ Dual use research of concern
☒	☐ Plants

Methods

n/a	Involved in the study
☒	☐ ChIP-seq
☐	☒ Flow cytometry
☒	☐ MRI-based neuroimaging

Antibodies

Antibodies used

ARG2 (A19233, Abclonal, U.S.A), ODC1 (A3898, Abclonal), GPX4 (DF6701, Affinity, U.S.A), ACSL4 (abs106075, Absin, China), SLC7A11 (DF12509, Affinity), GAPDH (AF0006, Beyotime), PAOX (abs139256, Absin), SMOX (abs151305, Absin), catalase (A18018, abclonal), c-Myc (A1309, Abclonal), TSG101 (A1692, Abclonal), CD63 (abs149061, Absin), GM130 (A5344, Abclonal), CD81 (1:1000, A4863, Abclonal).

Validation

All antibodies used in our study have been validated and detailed information could be found on the website from manufactures as listed below. Some of them have also been validated by our experiments as shown in this manuscript using either over-express or knockout strategies.
ARG2 (A19233, Abclonal, U.S.A), https://abclonal.com.cn/catalog/A19233.
Species: Rabbit. Application: WB, IHC-P.
Ref: Porous microneedle patch with sustained delivery of extracellular vesicles mitigates severe spinal cord injury. Nature communications.
Seq:
FDPTLAPATGTPVVGGLTYREGMYIAEEIHNTGLLSALDLVEVNPQLATSEEEAKTTANLAVDVIASSFGQTREGGHIVYDQLPTPSSPDESENQARVRI

ODC1 (A3898, Abclonal), https://abclonal.com.cn/catalog/A3898
Species: Mouse. Application: WB, ICC, IF.
Seq:MNNFGNEEFDCHFLDEGFTAKDILDQKINEVSSSDDKDAFYVADLGDILKKHLRWLKALPRVTPFYAVKCNDSKAIVKTLAATGTGFDCASKTEIQLVQ

GAPDH (AF0006, Beyotime), https://www.beyotime.com/product/AF0006.htm
Species: Rabbit. Application: WB.
Seq: Not provided.

GPX4 (DF6701, Affinity), http://www.affbiotech.com/goods-5503-DF6701-GPX4_Antibody.html
Species: Rabbit. Application: WB, IHC, IF/ICC.
Seq: Not provided.
Ref: Chen K et al. Cell Membrane Camouflaged Metal Oxide–Black Phosphorus Biomimetic Nanocomplex Enhances Photo-chemo-dynamic Ferroptosis. ACS Applied Materials & Interfaces 2022 Jun 06.

ACSL4 (abs106075, Absin), https://www.absin.cn/rabbit-acsl4-facl4-polyclonal-antibody/abs106075.html
Species: Rabbit. Application: WB, IHC, IF.
Seq: Not provided.
Ref: Retinol saturase mediates retinoid metabolism to impair a ferroptosis defense system in cancer cells. Cancer Research.

SLC7A11 (DF12509, Affinity), http://www.affbiotech.com/goods-15599-DF12509-xCT_Antibody.html
Species: Rabbit. Application: WB, IHC, IF/ICC.
Seq: Not provided.
Ref: He Q et al. Biochanin A protects against iron overload associated knee osteoarthritis via regulating iron levels and NRF2/System xc-/GPX4 axis. Biomedicine & Pharmacotherapy.

PAOX (abs139256, Absin), https://www.absin.cn/rabbit-paox-polyclonal-antibody/abs139256.html
Species: Rabbit. Application: WB, Elisa, IF/ICC.
Seq: Not provided.

SMOX (abs151305, Absin), https://www.absin.cn/anti-smox-antibody/abs151305.html
Species: Rabbit. Application: WB.
Seq: Not provided.

catalase (A18018, abclonal), https://abclonal.com.cn/catalog/A18018
Species: Rabbit. Application: WB, IP, Elisa.
Seq:
MADSRDPASDQMQHWKEQRAAQKADVLTTGAGNPVGDKLNVITVGPRGPLLVQDVVFTDEMAHFDRERIPERVVHAKGAGAFGYFEVTHDITKYSKA
KVFEHIGKKTPIAVRFSTVAGESGSADTVRDPRGFAVKFYTEDGNWDLVGNNTPIFFIRDPILFPSFIHSQKRNPQTHLKDPDMVWDFWSLRPESLHQVSFL
FSDRGIPDGHRHMNGYGSHTFKLVNA.

c-Myc (A1309, Abclonal), https://abclonal.com.cn/catalog/A1309
Species: Rabbit. Application: WB, Elisa.
Seq: LRQISNNRKCTSPRSSDTEENVKRRTHNVLERQRRNELKRSFFALRDQIPELENNEKAPKVVILKKATAYILSVQAEEQKLISEEDLLRKRRE
Ref: CD36 inhibits β-catenin/c-myc-mediated glycolysis through ubiquitination of GPC4 to repress colorectal tumorigenesis. Nature communications.

TSG101 (A1692, Abclonal), https://abclonal.com.cn/catalog/A1692
Species: Rabbit. Application: ELISA,WB,IF/ICC.
Seq: Not provided.

CD63 (abs149061, Absin), https://www.absin.cn/anti-cd63-antibody/abs149061.html
Species: Rabbit. Application: WB, IHC.
Seq: Not provided.

GM130 (A5344, Abclonal), https://abclonal.com.cn/catalog/A5344
Species: Rabbit. Application: WB, IHC-P, IF/ICC, Elisa.
Seq:
SKLAAAKKKLREYQQRNSPGVPTGAKKKKKIKNGSNPETTTSGGCHSPEDTPKDNAATLQPSDDTVLPGGVPSPGASLTSMAASQNHDADNVPNLMDETKTFSSTESLRQLSQQLNGLVCESATCVNGEGPASSANLKDLESRYQQLAVALDSSYVTNKQLNITIEKLKQQNQEITDQLEEEKKECHQKQGALREQLQVHIQTIGILVSEKAELQTALAHTQHAARQKEGESEDLASRLQYSRRRVGELERALSAVSTQQKKADRYNKELTKERDALRLEL.
Ref: RAB21 controls autophagy and cellular energy homeostasis by regulating retromer-mediated recycling of SLC2A1/GLUT1. Autophagy.

CD81 (1:1000, A4863, Abclonal), https://abclonal.com.cn/catalog/A4863
Species: Rabbit. Application: WB.
Seq:
ILFACEVAAGIWGFVNKDQIAKDVKQFYDQALQQAVVDDDANNAKAVVKTFHETLDCCGSSTLTALTTSVLKNNLCPSGSNIISNLFKEDCHQKIDDLFSG.
Ref: Discovery and Characterization of 1H-1,2,3-Triazole Derivatives as Novel Prostanoid EP4 Receptor Antagonists for Cancer Immunotherapy. Journal of medicinal chemistry

Eukaryotic cell lines

Policy information about cell lines and Sex and Gender in Research

Cell line source(s)	The human non-small cell lung cancer cell lines A549, H1299, PC9, H23, fibrosarcoma cell line HT1080, and human embryonic kidney cell line HEK-293T were purchased from the Chinese Academy of Science Cell Bank.
Authentication	The cell lines were authenticated by short tandem repeat profiling in 2023 and were passaged every 3 to 5 days according to cells' proliferating rates.
Mycoplasma contamination	All cell lines tested negative for mycoplasma contamination.
Commonly misidentified lines (See ICLAC register)	HEK-293T cells were used for luciferase reporter assay.

Animals and other research organisms

Policy information about studies involving animals; ARRIVE guidelines recommended for reporting animal research, and Sex and Gender in Research

Laboratory animals	Four-week-old male homozygous (Foxn1nu) mut/mut BALB/c nude mice were purchased from the GemPharmatech (Nanjing, China) and housed under specific-pathogen-free conditions with a 12 h light–12 h dark cycle. The ambient temperature was 21–23 °C, with 45% humidity and the mice had ad libitum access to water and standard chow (LabDiet, #5053).
Wild animals	No wild animals involved in this study.
Reporting on sex	The findings were not applied to only one sex.
Field-collected samples	This study did not involve samples collected from field.
Ethics oversight	All animal studies were conducted in compliance with the policies of the animal ethics committee of Zhongshan Hospital, Fudan University (Approval No: B2022-180R).

Note that full information on the approval of the study protocol must also be provided in the manuscript.

Flow Cytometry

Plots

Confirm that:

- [x] The axis labels state the marker and fluorochrome used (e.g. CD4-FITC).
- [x] The axis scales are clearly visible. Include numbers along axes only for bottom left plot of group (a 'group' is an analysis of identical markers).
- [x] All plots are contour plots with outliers or pseudocolor plots.
- [ ] A numerical value for number of cells or percentage (with statistics) is provided.

Methodology

Sample preparation	Lipid peroxidation test: Cells were seeded on 12-well plates and incubated overnight. The next day, treated cells were harvested by trypsinization and washed with phosphate-buffered saline (PBS, Beyotime). Next, the cells were suspended in a fresh medium containing 4 µM BODIPY 581/591 C11 dye (Thermo Fisher, USA) at 37°C in a humidified 5% CO2 atmosphere. After 30 min of incubation, the cells were washed with PBS, and the lipid peroxidation levels were assessed.
Instrument	BD Accuri C6 with a 488-nm laser.
Software	The results were analyzed in FlowJo_v10 software.
Cell population abundance	At least 10000 cells were analyzed for each sample.
Gating strategy	Initial cell population gating (FSC-Area VS SSC-Area) was adopted to make sure debris was excluded, and then FSC-Area VS FSC-Height was adopted to make sure only single cell was used for analysis.

- [x] Tick this box to confirm that a figure exemplifying the gating strategy is provided in the Supplementary Information.
